# Coordination of oxysterol binding protein 1 and VAP-A/B modulates the generation of cholesterol and viral inclusion bodies to promote grass carp reovirus replication

**DOI:** 10.3389/fimmu.2024.1419321

**Published:** 2024-07-16

**Authors:** Jia Qi Li, Jie Zhang, Yang Chen, Tao Le, Ming Xian Chang

**Affiliations:** ^1^ Chongqing Key Laboratory of Conservation and Utilization of Freshwater Fishes, College of Life Sciences, Chongqing Normal University, Chongqing, China; ^2^ Key Laboratory of Aquaculture Disease Control, Ministry of Agriculture, Institute of Hydrobiology, Chinese Academy of Sciences, Wuhan, China; ^3^ University of Chinese Academy of Sciences, Beijing, China

**Keywords:** grass carp OSBP1, VAP-A/B, cholesterol, viral inclusion bodies, grass carp reovirus

## Abstract

Similar to other RNA viruses, grass carp reovirus, the causative agent of the hemorrhagic disease, replicates in cytoplasmic viral inclusion bodies (VIBs), orchestrated by host proteins and lipids. The host pathways that facilitate the formation and function of GCRV VIBs are poorly understood. This work demonstrates that GCRV manipulates grass carp oxysterol binding protein 1 (named as gcOSBP1) and vesicle-associated membrane protein–associated protein A/B (named as gcVAP-A/B), 3 components of cholesterol transport pathway, to generate VIBs. By siRNA-mediated knockdown, we demonstrate that gcOSBP1 is an essential host factor for GCRV replication. We reveal that the nonstructural proteins NS80 and NS38 of GCRV interact with gcOSBP1, and that the gcOSBP1 is recruited by NS38 and NS80 for promoting the generation of VIBs. gcOSBP1 increases the expression of gcVAP-A/B and promotes the accumulation of intracellular cholesterol. gcOSBP1 also interacts with gcVAP-A/B for forming gcOSBP1-gcVAP-A/B complexes, which contribute to enhance the accumulation of intracellular cholesterol and gcOSBP1-mediated generation of VIBs. Inhibiting cholesterol accumulation by lovastatin can completely abolish the effects of gcOSBP1 and/or gcVAP-A/B in promoting GCRV infection, suggesting that cholesterol accumulation is vital for gcOSBP1- and/or gcVAP-A/B-mediated GCRV replication. Thus, our results, which highlight that gcOSBP1 functions in the replication of GCRV via its interaction with essential viral proteins for forming VIBs and with host gcVAP-A/B, provide key molecular targets for obtaining anti-hemorrhagic disease grass carp via gene editing technology.

## Highlights

- Grass carp OSBP1 and VAP-A/B promote GCRV replication
- Grass carp OSBP1 is recruited by NS38 and NS80 of GCRV
- Grass carp OSBP1 promotes the generation of intracellular cholesterol and VIBs
- Grass carp OSBP1 interacts with VAP-A/B
- Grass carp OSBP1 collaborates with VAP-A/B to promote GCRV replication

## Introduction

Lipid-binding/transfer proteins (LTPs) function in transferring lipids from one membrane to another in the vesicle-independent manner. In mammals, oxysterol binding proteins (OSBPs) are one of the highly conserved LTP families and encoded by 12 genes, which are categorized into I-VI six subfamilies (1). Most of them have a pleckstrin homology (PH) domain binding phosphoinositides or a short amino acid motif (FFAT) for interaction with vesicle-associated membrane protein–associated protein A/B (VAP-A/B) on the cytoplasmic surface of the endoplasmic reticulum (ER), and a conserved OSBP-related ligand-binding domain (LBD or ORD) (1, 2). In the case of OSBP1, the LBD binds cholesterol and its catabolic derivative 25-hydroxycholesterol (25OH). OSBP1 exists in the cytoplasm and ER when bound to cholesterol alone, however translocates to the Golgi apparatus when bound to 25OH or removal of cholesterol (3). The dysfunctions of OSBPs in cholesterol transport are involved in many diseases, including fatty liver disease, cardiometabolic diseases, cancer and so on (4, 5).

Viruses have been reported to exploit host cell membranes and its lipids for creating optimal conditions to support the viral life cycle via invading cells, replicating, assembling new virions, and eventually being released from the host cells (1). Positive-strand RNA viruses, such as Hepatitis C virus (HCV), encephalomyocarditis virus (EMCV) and enterovirus, have the ability to regulate intracellular lipid balance for forming membrane-structured “viral replication organelles” (ROs) and complete the viral genome replication process within these specialized structures. OSBP, which exchanges cholesterol and PI4P between ER and Golgi at membrane contact sites (MCS) or transfers cholesterol to the ROs, is required for the replication of positive-strand RNA viruses (6, 7). The nonstructural protein NS5A of HCV has been shown to interact with OSBP at the Golgi apparatus, which is essential for the viral assembly and secretion (8). Of note, itraconazole and posaconazole were revealed to target OSBP to inhibit HCV replication (9, 10). Furthermore, OSBP has also engaged in HCV and poliovirus (PV) replication and infection, but dispensable for dengue virus (DENV) (11). Therefore, the functions of OSBPs seem to be variable dependent on virus type.

Grass carp reovirus (GCRV), which causes severe hemorrhagic disease in grass carp (*Ctenopharyngodon idella*), is a member of the Reoviridae. Like other DNA or RNA viruses, GCRV replicates their genomic RNA within specialized intracellular compartments named viral inclusion bodies (VIBs, also named ROs or viroplasms). The NS80 and NS38 of GCRV-873 (one of the GCRV-I genotype) are two main proteins to form VIBs, which help the virus to carry out the replication and assembly process more efficiently by recruiting viral and host factors into VIBs (12, 13). For GCRV-II, the VP56 and VP35 induce the formation of VIBs, and recruit other viral proteins or RNAs to VIBs for viral replication (14). Recently, we revealed that several host factors regulated the generation of VIBs. The NS80 and NS38 of GCRV-873 can hijack piscine TBK1 into the VIBs to avoid host antiviral immune response, however host can generate TBK1_tv3 isoform via alternative splicing to degrade the NS80 and NS38 for impairing the generation of GCRV VIBs (15, 16). Further studies on the components and regulators of GCRV VIBs will help to provide anti-viral drug targets via targeting the life cycle of viruses.

In teleost, 14 orthologs, including osbp, osbp2, osbpl1a, osbpl2a, osbpl2b, osbpl3a, osbpl3b, osbpl5, osbpl6, osbpl7, osbpl8, osbpl9, osbpl10 and osbpl11, are identified in zebrafish, and locate in 11 different chromosomes. The organization of functional domains of OSBPs is highly conserved between zebrafish and human (2). Several studies reveal the role of zebrafish OSBPL2 in cholesterol biosynthesis, ROS production, hearing loss and development (17, 18). However, the function of other piscine OSBPs, especially during pathogen infection, is still unclear. Here, we identified grass carp OSBP1 (named as gcOSBP1) as a key player in the replication of GCRV. The NS80 and NS38 proteins of GCRV interact with and recruit gcOSBP1 to VIBs which increases the cholesterol concentration and leads downstream recruitment of gcVAP-A/B. Our data reveal that the production of cholesterol is important for the negative regulation of gcOSBP1 and collaboration between gcOSBP1 and gcVAP-A/B in promoting the generation of VIBs.

## Materials and methods

### Abs and reagents

The anti-FLAG mouse mAb (#F3165), anti-HA rabbit mAb (#51064-2-AP) and anti-GAPDH mouse mAb (#60004-1-Ig) were purchased from Sigma-Aldrich and Proteintech, respectively. The anti-VP3, anti-VP5, anti-NS80 and anti-NS38 polyclonal rabbit Abs against the GCRV-873 strain were prepared previously (19). Goat anti-mouse Ig-HRP conjugate secondary Ab, goat anti-rabbit Ig-HRP conjugate secondary Ab, Alexa Fluor 594 conjugated secondary Ab against rabbit IgG, Alexa Fluor 488 conjugated secondary Ab against mouse IgG, 4´,6-diamidino-2-phenylindole (DAPI), RevertAid First Stand cDNA Synthesis Kit (#K1622), Lipofectamine 2000, and protease inhibitor mixture were purchased from Thermo Fisher Scientific. The FLAG^®^ Immunoprecipitation Kit and Dimethylsulfoxide (DMSO) were purchased from Sigma-Aldrich. Trizol reagent (#15596026) was purchased from Invitrogen. Total cholesterol assay kit (#A111-1-1) was purchased from Nanjing Jiancheng. Lovastatin (#S2061) was purchased from Selleck.

### Cells, virus and plasmids

CIK (*Ctenopharyngodon idellus* kidney) cells were grown in minimum essential medium (MEM) supplemented with 10% FBS. Grass carp reovirus (GCRV-873) was propagated in CIK cells using MEM supplemented with 2% FBS. The GCRV titer was quantified by the TCID_50_ method. CIK cells were seeded in 96-well plates (1×10^5^ cells/well). For each sample, 6 replicate wells were infected with different dilutions separated by a 10-fold dilution. After 1 h, the inoculated viral suspensions were removed. Then the cells were washed with PBS, and incubated in 100 μL MEM medium containing 2% FBS for 2 or 3 d. Given that GCRV-873 can produce obvious cytopathic effect (CPE), the wells with CPE were recorded. According to the Reed-Muench formula, the TCID_50_ of GCRV was calculated, and then converted into PFU/mL (PFU/mL = 0.7 × TCID_50_/mL).

Plasmids used in this study including pTurbo-GFP vector (Evrogen), pcDNA3.1-FLAG-HA vector (Invitrogen) and p3×FLAG-CMV™-14 Expression Vector (Sigma-Aldrich). gcOSBP1-FLAG, gcOSBP1-GFP and gcOSBP1-HA were obtained using the primer pairs gcOSBP1-F/gcOSBP1-R or gcOSBP1-F1/gcOSBP1-R1 or gcOSBP1-F2/gcOSBP1-R2, and cloned into the p3×FLAG-CMV-14 or pTurbo-GFP or pcDNA3.1-FLAG-HA vector, respectively. gcVAP-A-FLAG and gcVAP-B-FLAG were obtained using the primer pairs gcVAP-A-F/gcVAP-A-R and gcVAP-B-F/gcVAP-B-R, and cloned into the p3×FLAG-CMV™-14 vector. gcVAP-A-HA and gcVAP-B-HA were obtained using the primer pairs gcVAP-A-F1/gcVAP-A-R1 and gcVAP-B-F1/gcVAP-B-R1, and cloned into the pcDNA3.1-FLAG-HA vector. The primers used for plasmid constructs are listed in **Table 1**.

**Table 1:** Primers used for the present study.

Primers	Sequences (5’-3’)	Application
gcOSBP1-F	TTGCGGCCGCGATGTCGGAGCCC AAAG	Ligated to p3xFLAG-CMV™-14 vector
gcOSBP1-R	CGGGATCCGAAGATATCCGGGC AGGAG	
gcVAP-A-F	GGAATTCAATGTCCAAACTGGAGCAG	
gcVAP-A-R	GGGGTACCAACAAGACAAACTTCCCT AGGAAG	
gcVAP-B-F	GGAATTCAATGGCCAGGCCAGAG	
gcVAP-B-R	GGGGTACCAACAAGGCCAACTTGCCG	
gcOSBP1-F1	CCCAAGCTTATGTCGGAGCCCAAAG	Ligated to pTurboGFP-N vector
gcOSBP1-R1	CGGGATCCAAGAAGATATCCGGGCA	
gcOSBP1-F2	GGGGTACCATGTCGGAGCCCAAAG	Ligated to pcDNA3.1-FLAG-HA vector
gcOSBP1-R2	GCTCTAGAGAAGATATCTGGGCAGG	
gcVAP-A-F1	GGAATTCATGTCCAAACTGGAGCAG	
gcVAP-A-R1	GGGGTACCCAAGACAAACTTCCCTA GGAAG	
gcVAP-B-F1	GGAATTCATGGCCAGGCCAGAG	
gcVAP-B-R1	GGGGTACCCAAGGCCAACTTGCCG	
si-gcOSBP1-1	TCACTACACCTGGAAGAAA	siRNAs
si-gcOSBP1-2	AGACTCTCAAAGCCAAAGA	
si-gcOSBP1-3	CGAATTACTCTCTCAATCT	
q-β-actin-F	TCTTGGGTATGGAGTCTTGCG	qRT-PCR
q-β-actin-R	TTGATTTTCATTGTGCTAGGGGC	
q-EF1α-F	CTGGAACCTCACAGGCAGAC	
q-EF1α-R	GCTGCTTCACACCCAATGTG	
q-18S-F	ATTTCCGACACGGAGAGG	
q-18S-R	CATGGGTTTAGGATACGCTC	
q-gcOSBP1-F	GTTCGGCTGGAAGGACAGACAA	
q-gcOSBP1-R	GCGTTGGAGGTGATACGGAAGA	
q-gcVAP-A-F	AAGGTCTGAGGATGAGGAAGGC	
q-gcVAP-A-R	ATGAAGATGGCGGCGATGAC	
q-gcVAP-B-F	AGCGGCTACGAGAGGAGAACAA	
q-gcVAP-B-R	ATGAGTGCGGAGCGGACATTAC	
q-NS38-F	CTATGGCACTGGCGTTTA	
q-NS38-R	GTCGGGTAGTTCAGAGGG	
q-NS80-F	GGAAGCCGACAAGGGAATG	
q-NS80-R	TGGAGTAGCCGTGGGAAG	

### Sequence analysis

Protein structure prediction was used via SMART version 4.0 software. Meanwhile, protein conserved domains were predicted by the conserved domain database analysis of NCBI and mapped by IBS software. Multiple alignments of amino acid sequences were performed by ClustalW programs. The phylogenetic analysis was constructed using MEGA 11 with Neighbor-Joining method.

### Knockdown of gcOSBP1 by small interfering RNA

The siRNA technology was used to knockdown of gcOSBP1. Three siRNA sequences of gcOSBP1 (**Table 1**) were synthesized by RiboBio company (Guangzhou, China). CIK cells (6-well plates, 1 × 10^6^ per well) were transfected with 100 nM siRNA targeting the gcOSBP1 or with siRNA-control, respectively. Twenty-four hours after transfection, these cells were collected for qRT-PCR to detect the transcription of gcOSBP1 for determining the silencing effects of these siRNAs.

### Viral infection assays

To reveal the function of gcOSBP1 overexpression or knockdown in viral infection, CIK cells (24-well plates, 2.5 × 10^5^ per well) were transfected with 500 ng indicated plasmids or 100 nM indicated siRNA, respectively. After 24 h, these cells were treated with GCRV at an indicated multiplicity of infection (MOI). Lovastatin is a cell-permeable HMG-CoA reductase inhibitor used to reduce cholesterol synthesis. To explore the possible role of impairing cholesterol synthesis on the gcOSBP1- and/or gcVAP-A/B-mediated GCRV replication, cells (24-well plates, 2.5 × 10^5^ per well) were transfected with 500 ng indicated plasmid for a single plasmid transfection or 250 ng indicated plasmids for co-transfection of two plasmids for 22 h, added lovastatin (5 μM) and incubated for 2 h. Then, these cells were exposed to GCRV (MOI = 1). To determine the function of gcVAP-A/gcVAP-B and gcOSBP1-gcVAP-A/gcVAP-B interaction in GCRV infection, 250 ng indicated plasmids were co-transfected into CIK cells (24-well plates, 2.5 × 10^5^ per well). After 24 h, GCRV (MOI = 1) infected these cells. Following viral adsorption for 1 h, the above-mentioned cells were washed and maintained in MEM with 2% FBS. At 24 hours post infection (hpi), the supernatants were collected for detecting viral titers. The cells were fixed with 4% PFA, stained by 1% crystal violet, and lastly photographed.

### Measurement of total cholesterol

CIK cells seeded overnight in 6-well plates were transfected with indicated plasmids. After 24 h post-transfection, these cells were infected with GCRV at a MOI of 1. At 24 hpi, the cells were collected and suspended in 100 μL PBS buffer. Ultrasonication was performed on a 950E ultrasonic homogenizer (SCIENTZ). Total cholesterol content was detected by T-CHO assay kit. The absorbance at 500 nm was measured by a microplate reader (Synergy™ Neo2 Multi-Mode Microplate Reader, Bio-Tek, USA).

### Immunofluorescence assays

To determine subcellular localization of gcOSBP1 in Golgi apparatus, CIK cells (24-well plates, 2.5 × 10^5^ per well) were transfected with 500 ng indicated plasmid for 36 h, and then left untreated or exposed to GCRV (MOI = 1) for 24 h. After washing with PBS, the cells were added the Golgi-Tracker Red working solution and incubated at 4°C for 30 minutes. After washing with 2% MEM, the cells were added 2% MEM and incubated at 37°C for another 30 minutes. To determine whether gcOSBP1 localized in VIBs, a similar transfection protocol was performed in CIK cells. After 36 hours of transfection with either the empty GFP plasmid or gcOSBP1-GFP, the cells were exposed to GCRV (MOI = 1) for 24 h. Subsequently, the infected cells were fixed with 4% PFA for 1 h at 25°C. After 15 mins of 1‰ triton X-100 permeabilization and 1 h 5% BSA blocking, the cells were incubated with rabbit anti-NS38 (1:500) for 2 h, and followed by incubation with Alexa Fluor 594 conjugated secondary Ab against rabbit IgG (Thermo Fisher Scientific, 1:500). To further investigate the roles of gcOSBP1 and gcOSBP1-gcVAP-A/gcVAP-B interaction on the generation of GCRV VIBs, CIK cells (24-well plates, 2.5 × 10^5^ per well) were transfected with 100 nM siRNA targeting the gcOSBP1 or with siRNA-control, 500 ng FLAG or gcOSBP1-FLAG, or with indicated combination of various plasmids for 36 h. Then, the following steps as above. Hoechst 33342 stained the cell nucleus for 20 min and a confocal microscope was used to take a picture.

### Co-immunoprecipitation assay and western blotting

To clear the potential relationship between gcOSBP1 and viral proteins or gcVAP-A/gcVAP-B, CIK cells were transfected with 8 μg indicated plasmids for 36 h, and then exposed to GCRV (MOI = 1) for another 12 h or remained untreated. To determine the potential interactions between gcOSBP1 and gcVAP-A/gcVAP-B, CIK cells were transfected with indicated plasmids for 48 h. All these samples were lysed in IP lysis buffer. Co-IP was performed according to the previously reported methods (19, 20). Western blotting used the following antibodies: anti-FLAG (Sigma-Aldrich, 1:5000), anti-HA (Sigma-Aldrich, 1:5000), anti-GAPDH (Proteintech, 1:5000), anti-VP3 (1:1000), anti-VP5 (1:5000), anti-NS38 (1:5000), or anti-NS80 (1:5000) Abs. The protein ratio of bands was quantified by Quantity One.

### RNA extraction, reverse transcription and qRT-PCR

To determine the expression of gcOSBP1 regulated by GCRV infection, CIK cells (6-well plates, 1 × 10^6^ per well) were treated with GCRV (MOI = 1) and collected at 6, 12 and 24 hpi, and then used for RNA extraction. To determine the effects of gcOSBP1 on the expression of gcVAP-A/gcVAP-B and NS38/NS80, CIK cells were transfected with FLAG or gcOSBP1-FLAG for 22 h, and next treated with lovastatin (5 μM) for 2 h. Subsequently, these cells were exposed to GCRV (MOI = 1). At 24 hpi, the cells were used for RNA extraction. The qRT-PCR was performed according to our previously reported method (20). All primers used for qRT-PCR are listed in **Table 1**.

### Statistical analysis

Statistical analysis and graphs were performed and produced using GraphPad Prism 7.0 software. Data from qRT-PCR are presented as mean and SEM. The significance of results was analyzed by an ANOVA or Student’s t-test (*p < 0.05, **p < 0.01, ns, not significant).

## Results

### Sequence and evolutionary tree analysis of gcOSBP1

**Figure 1:**
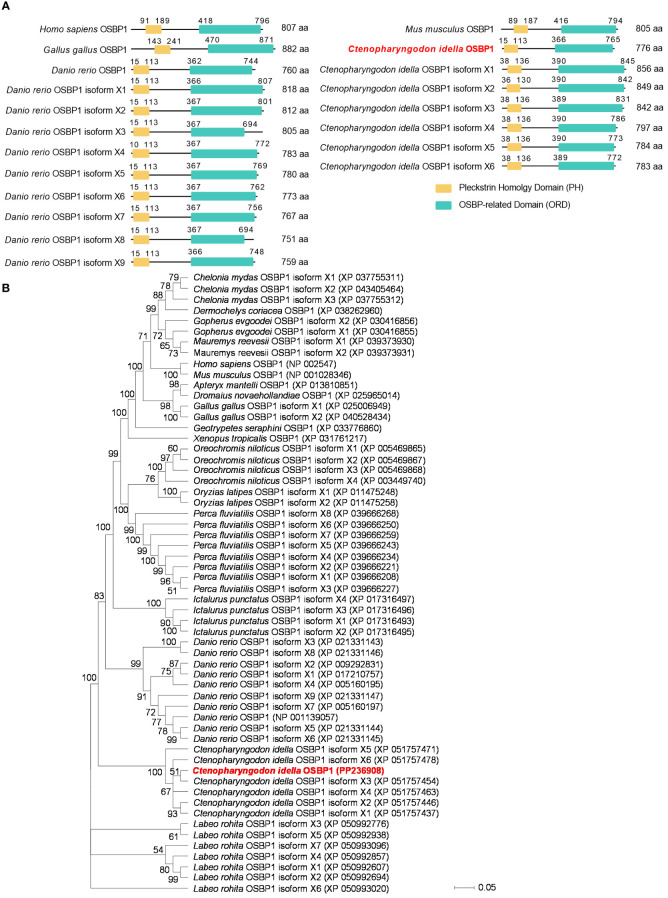
Domain and evolutionary tree analysis of vertebrate OSBP1. **(A)** Domain analysis of vertebrate OSBP1. **(B)** Phylogenetic trees of vertebrate OSBP1 were constructed using MEGA 11 with the neighbor-joining method. Bootstrap values for 1 000 replicates (%) are indicated on each branch.

Six gcOSBP1 isoforms exist in grass carp database. The isoform X1 of gcOSBP1 (GenBank accession number: XP_051757437) encodes 856 aa, 849 aa for isoform X2 of gcOSBP1 (GenBank accession number: XP_051757446), 842 aa for isoform X3 of gcOSBP1 (GenBank accession number: XP_051757454), 797 aa for isoform X4 of gcOSBP1 (GenBank accession number: XP_051757463), 784 aa for isoform X5 of gcOSBP1 (GenBank accession number: XP_051757471) and 783 aa for isoform X6 of gcOSBP1 (GenBank accession number: XP_051757478). The gcOSBP1 we obtained from grass carp (GenBank accession number: PP236908) encodes 776 aa (**Figure 1**). Similar to OSBP1 from the mammals, aves and teleosts, all the OSBP1s contain a N-terminal PH domain and a C-terminal ORD domain (**Figure 1A**). To further confirm the identities of the gcOSBP1 we obtained and other OSBP1s from the mammals, aves and teleosts, a phylogenetic tree was constructed using the neighbor-joining method based on the alignments of all the OSBP1s. The gcOSBP1 we obtained is closer with isoform X3 of gcOSBP1, and formed a single branch with 6 gcOSBP1 isoforms from grass carp database (**Figure 1B**).

### gcOSBP1 is required for GCRV replication

**Figure 2:**
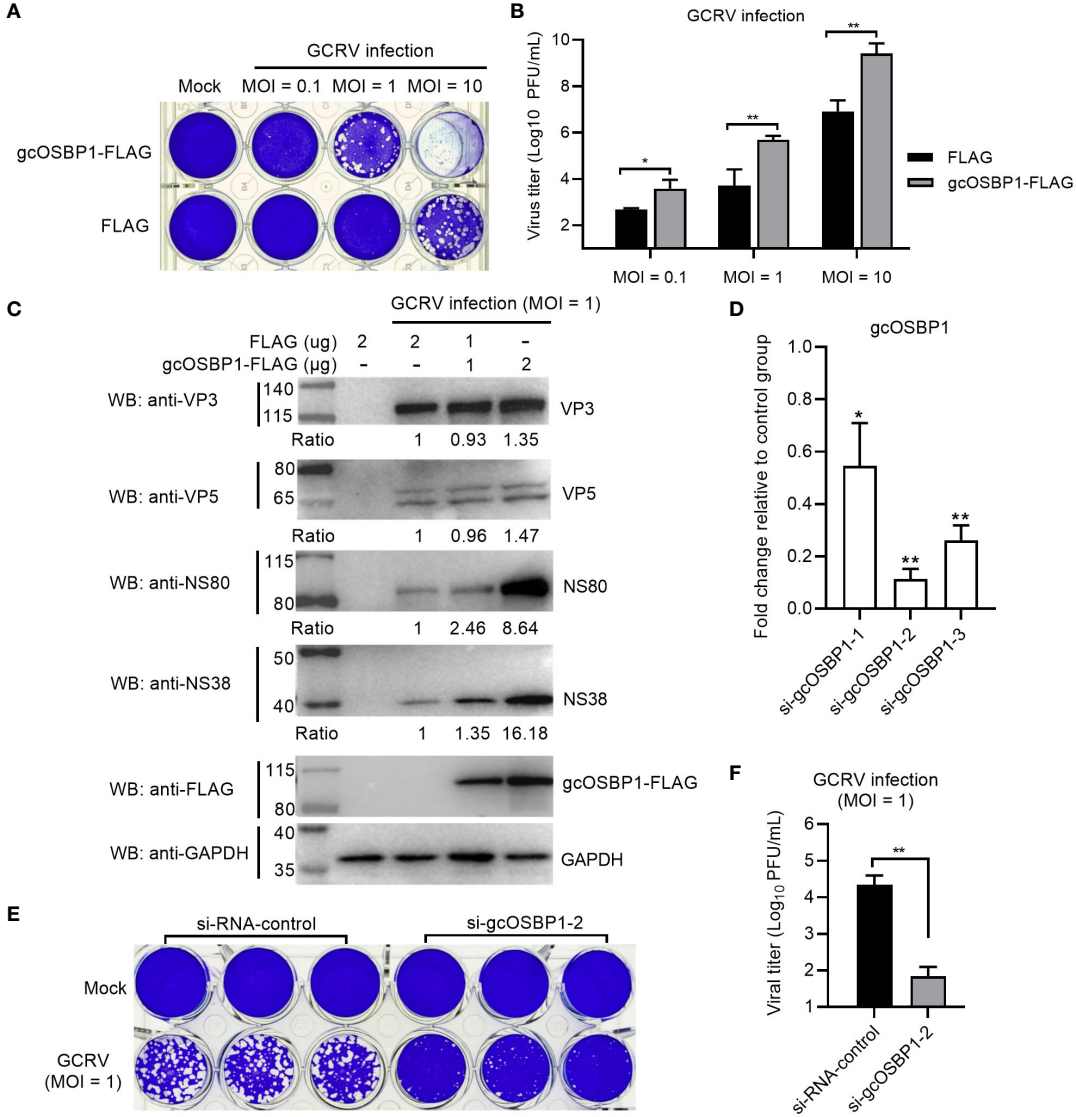
The effect of gcOSBP1 on the GCRV replication. **(A, B)** Crystal violet staining **(A)** and determination of GCRV titers **(B)** for overexpression of gcOSBP1 in CIK cells infected with GCRV at the MOI of 0.1, 1 and 10. **(C)** The effect of gcOSBP1 on the expressions of GCRV proteins including VP3, VP5, NS38 and NS80 in CIK cells infected with GCRV at the MOI of 1. Protein bands were quantified by Image J. **(D)** The effect of knockdown of gcOSBP1 on the expression of gcOSBP1 in CIK cells transfected with si-gcOSBP1-1, si-gcOSBP1-2 or si-gcOSBP1-3. **(E, F)** Crystal violet staining **(E)** and determination of GCRV titers **(F)** for knockdown of gcOSBP1 in CIK cells infected with GCRV at the MOI of 1. For **(B, F)**, the asterisk above the bracket indicated statistical significance between the two groups connected by the bracket. For **(D)**, the asterisk above the error bars indicated statistical significance using the group transfected with si-RNA-control as the control group. *p < 0.05, **p < 0.01.

To investigate whether gcOSBP1 might be involved in GCRV replication, we transfected CIK cells with FLAG or gcOSBP1-FLAG. Compared with the FLAG empty plasmid, overexpression of gcOSBP1 significantly elevated the susceptibility of CIK cells upon infection with GCRV at the MOI of 1 and 10 (**Figure 2A**). Virus replication was strongly increased upon gcOSBP1 overexpression at the MOI of 0.1, 1 and 10, as measured by TCID_50_ (**Figure 2B**). Overexpression of gcOSBP1 also induced the expression levels of VP3, VP5, NS38 and NS80 proteins, especially NS38 and NS80 with the increase of 16.18-fold and 8.64-fold respectively in the transfection dose of 2 μg (**Figure 2C**). To verify the role of gcOSBP1 for GCRV replication, we conducted knockdown experiments against gcOSBP1. qRT-PCR showed that the si-gcOSBP1-2 (100 nM) exhibited the most excellent silencing effect and consistency of experimental replicates (**Figure 2D**). Based on such results, we selected si-gcOSBP1-2 for subsequent experimental studies. We next investigated the effect of gcOSBP1 silencing on GCRV replication. The knockdown of gcOSBP1 inhibited GCRV infection and also reduced GCRV titers (**Figures 2E, F**). In conclusion, these results suggest the involvement of gcOSBP1 in GCRV replication.

### gcOSBP1 is induced by GCRV infection and localizes in the cytoplasm of infected cells including the Golgi complex and VIBs

**Figure 3:**
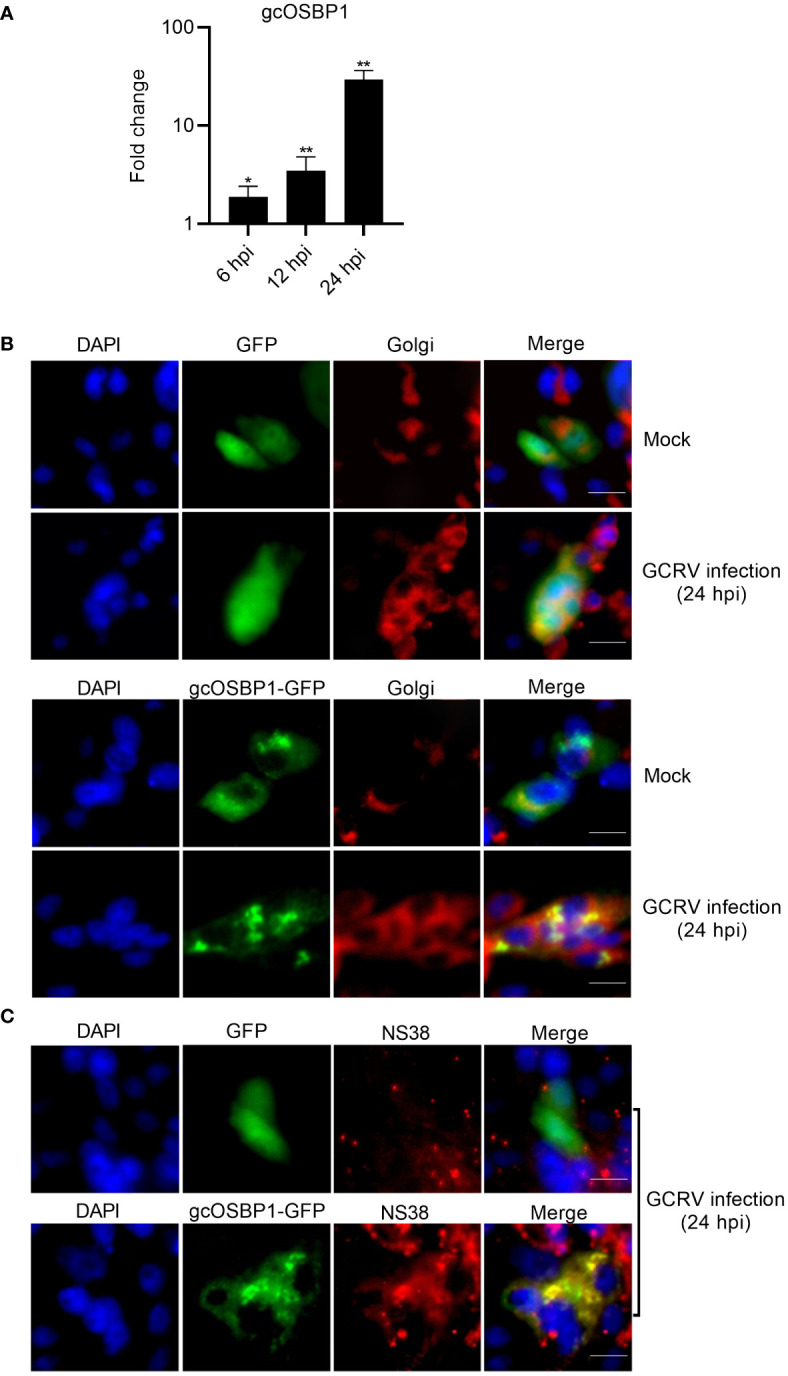
The expression and subcellular localization of gcOSBP1. **(A)** The expression of gcOSBP1 regulated by GCRV infection in CIK cells. Cell samples were collected at 6, 12 and 24 hpi, and used for qRT-PCR. The asterisk above the error bars indicated statistical significance using the uninfected group as the control group. *p < 0.05, **p < 0.01. **(B)** The subcellular co-localizations of gcOSBP1 and Golgi complex in CIK cells with or without the GCRV infection. Scale bars, 10 µm. **(C)** The subcellular co-localizations of gcOSBP1 and VIBs in CIK cells with the GCRV infection. Scale bars, 10 µm.

The expression of gcOSBP1 was firstly investigated in CIK cells infected with GCRV for 6, 12 and 24 h. The expression of gcOSBP1 were increased after infection with GCRV at all tested time points, with the continuous increase from 6 to 24 hpi (**Figure 3A**).

Mammalian OSBP1 exhibited subcellular localization in the cytoplasm and ER, shifting to exclusively Golgi after 25OH treatment (21). We postulated that GCRV infection might modulate the subcellular localization of gcOSBP1. To investigate this hypothesis, we used gcOSBP1-GFP plasmid and Golgi marker for live-cell immunofluorescence staining. CIK cells transfected with GFP empty plasmid showed the localization at the whole cell, and has no obvious change in response to GCRV infection. The Golgi apparatus presents a compact structure in the absence of infection, but scattered by GCRV infection. Under normal conditions, gcOSBP1 localized in the cytoplasm of cells, with a small amount localized at the Golgi complex. GCRV infection concentrated the subcellular localization of gcOSBP1 in discrete locations within the cytoplasm. The localization of gcOSBP1 at the Golgi complex was more obvious during GCRV infection (**Figure 3B**).

Due that gcOSBP1 significantly induces the expressions of NS38 and NS80 proteins that form VIBs, we next determine whether gcOSBP1 are actually present in VIBs. In the GFP empty plasmid group infected with the GCRV, the staining of NS38 was weak, and no co-localization was observed between GFP and NS38. Overexpression of gcOSBP1 increased the expression of NS38, and the obvious co-localization was observed between gcOSBP1-GFP and NS38 (**Figure 3C**).

These results collectively demonstrate that under conditions of GCRV infection, gcOSBP1 is induced and localizes in cytoplasm including the Golgi complex and VIBs.

### gcOSBP1 is recruited by NS38 and NS80 for promoting the generation of VIBs

**Figure 4:**
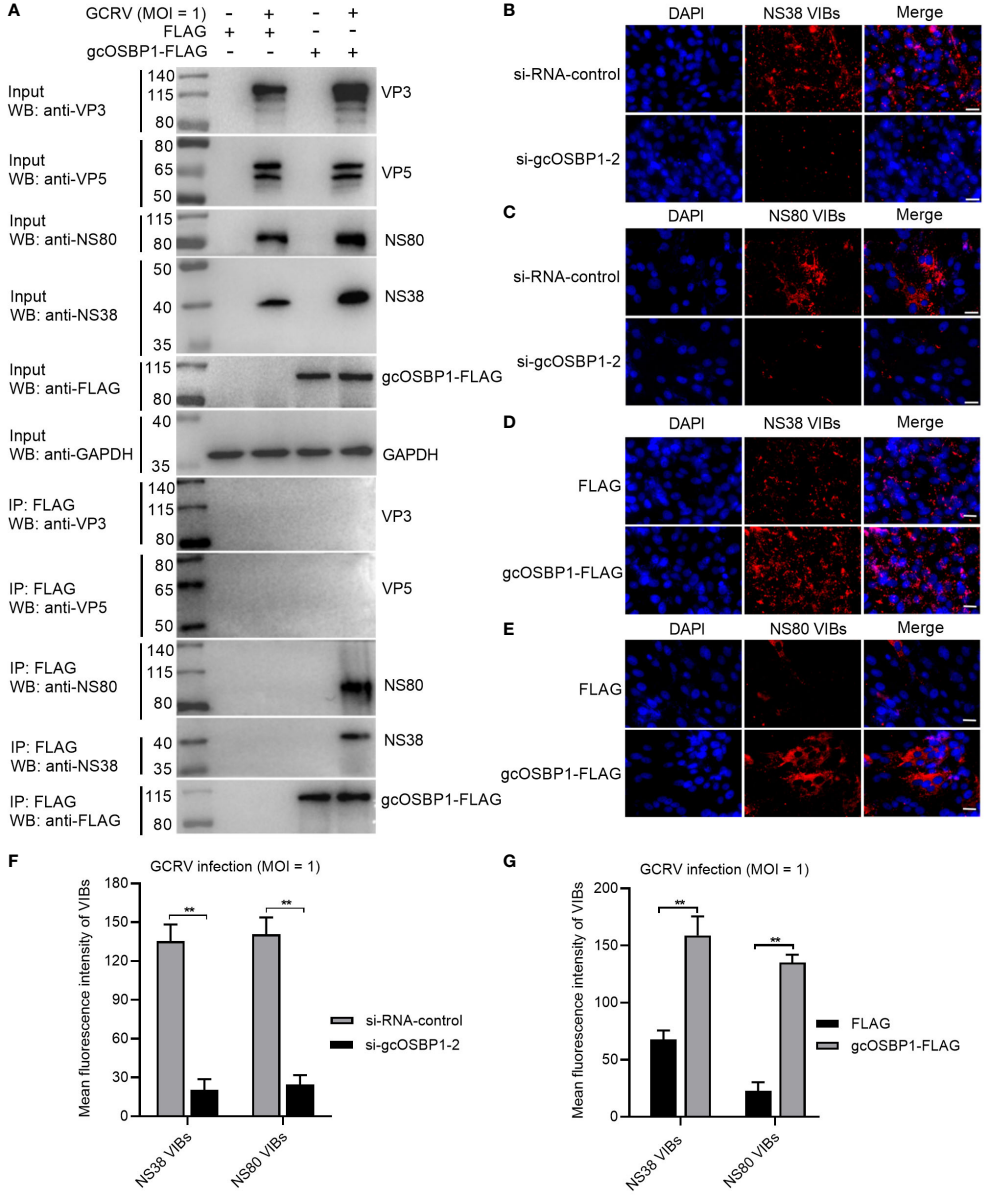
gcOSBP1 interacts with NS38 and NS80 of GCRV, and promotes the generation of VIBs. **(A)** The interaction between gcOSBP1 and GCRV proteins including VP3, VP5, NS38 and NS80. **(B, C)** The effect of knockdown of gcOSBP1 on the production of VIBs determined by anti-NS38 or NS80 antibody. Scale bars, 10 µm. **(D, E)** The effect of overexpression of gcOSBP1 on the production of VIBs determined by anti-NS38 or anti-NS80 antibody. Scale bars, 10 µm. **(F, G)** The average fluorescence intensity of VIBs in CIK cells with knockdown or overexpression of gcOSBP1. **p < 0.01.

Whether gcOSBP1 interacts with viral proteins is further investigated. Among 12 GCRV proteins, 2 nonstructural proteins (NS38 and NS80) forming GCRV VIBs and 2 structural proteins (VP3 and VP5) were chosen for the following research due that we have antibodies against VP3, VP5, NS80 and NS38 proteins of GCRV (19). Results of Co-IP revealed that gcOSBP1 could interact with NS38 and NS80 proteins, but not with VP3 and VP5 structural proteins of GCRV (**Figure 4A**). Next, we examined the effect of gcOSBP1 on the generation of GCRV VIBs. Using the anti-NS38 and anti-NS80 antibodies, we observed that the numbers of VIBs were significantly reduced by the knockdown of gcOSBP1 when compared with si-RNA-control, however increased by the overexpression of gcOSBP1 when compared with the control group transfected with FLAG (**Figures 4B, G**). These results indicate that gcOSBP1 is recruited by NS38 and NS80 proteins via protein-protein interaction for promoting the generation of VIBs.

### gcOSBP1 collaborates with gcVAP-A/B to promote GCRV replication

**Figure 5:**
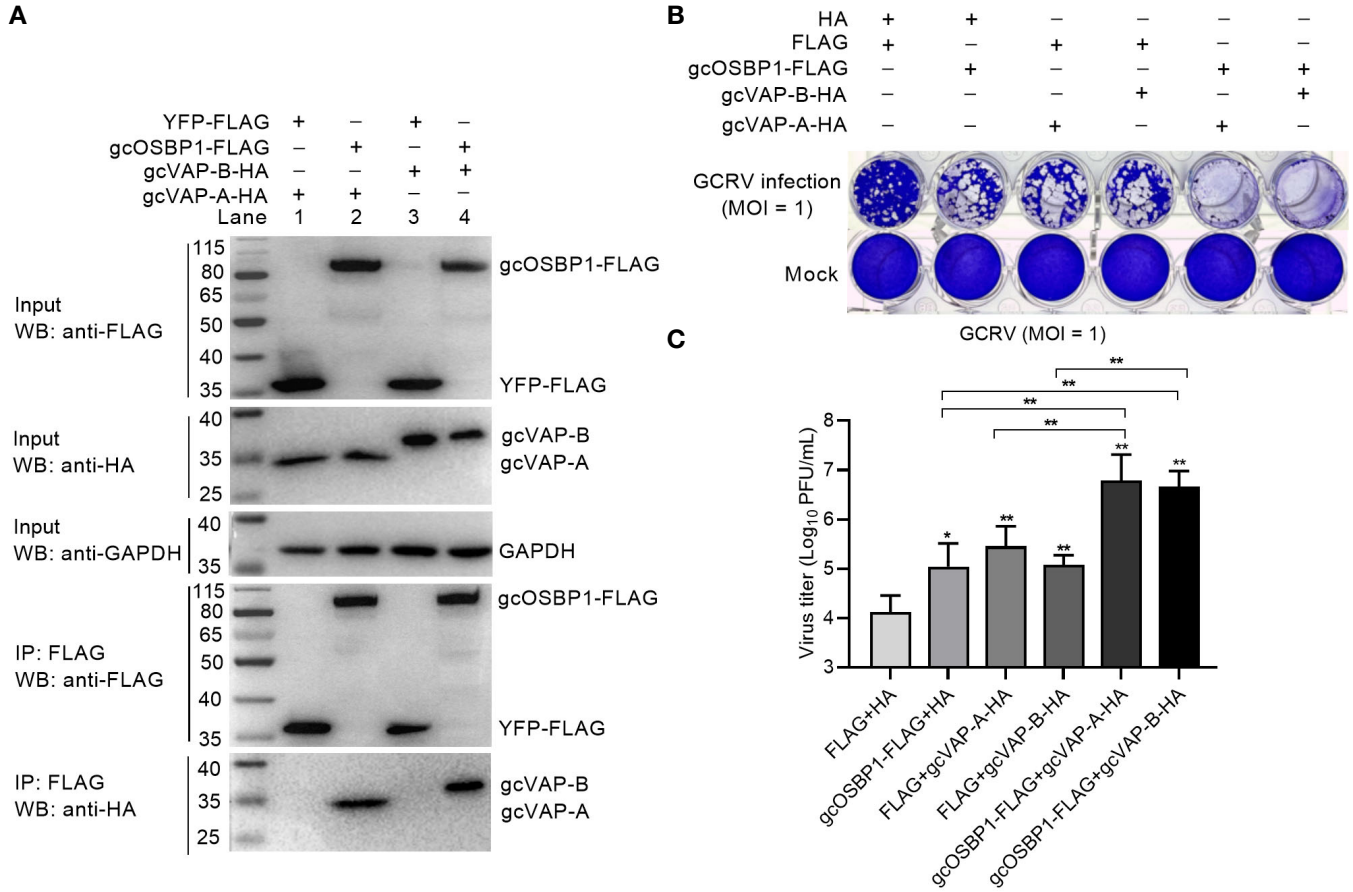
gcOSBP1 interacts with gcVAP-A/B to facilitate GCRV replication. **(A)** The interaction between gcOSBP1 and gcVAP-A/B. **(B, C)** Crystal violet staining **(B)** and determination of GCRV titers **(C)** for overexpression of gcOSBP1 and/or gcVAP-A/B in CIK cells infected with GCRV at the MOI of 1. For **(C)**, data are presented as mean values ± SD (n = 3). The asterisk above the error bars indicated statistical significance using the group transfected with FLAG plus HA as the control group. The asterisk above the bracket indicated statistical significance between the two groups connected by the bracket. *p < 0.05, **p < 0.01.

VAP-A and VAP-B are necessary for HCV replication, and can interact with homologs of OSBP (22, 23). We therefore examined whether gcOSBP1 interacted with gcVAP-A and gcVAP-B. Co-IP using FLAG-tagged gcOSBP1 and hemagglutinin (HA)-tagged gcVAP-A/B confirmed that gcOSBP1 was associated with gcVAP-A/B (**Figure 5A**). To further assess the effects of gcOSBP1-gcVAP-A/B interaction on GCRV infection, CIK cells expressing empty plasmids HA plus FLAG, HA plus gcOSBP1, FLAG plus gcVAP-A, FLAG plus gcVAP-B, gcOSBP1 plus gcVAP-A or gcOSBP1 plus gcVAP-B were infected with GCRV. Similar to gcOSBP1, overexpression of gcVAP-A or gcVAP-B significantly increased the susceptibility of cells to GCRV at the MOI of 1. Most notably, the cytopathic effects were more obvious in CIK cells expressing gcOSBP1 plus gcVAP-A or gcVAP-B compared to CIK cells expressing gcOSBP1, gcVAP-A or gcVAP-B alone (**Figure 5B**). Not surprisingly, replication of GCRV was moderately elevated in CIK cells expressing gcOSBP1 (8.54-fold), gcVAP-A (21.74-fold) or gcVAP-B (9.44-fold) alone, whereas dramatically elevated in CIK cells expressing gcOSBP1 plus gcVAP-A (552.9-fold) or gcOSBP1 plus gcVAP-B (360.2-fold). These results collectively demonstrate that gcOSBP1 collaborates with gcVAP-A/B to promote GCRV replication.

### gcOSBP1-mediated cholesterol accumulation is critical for GCRV replication

**Figure 6:**
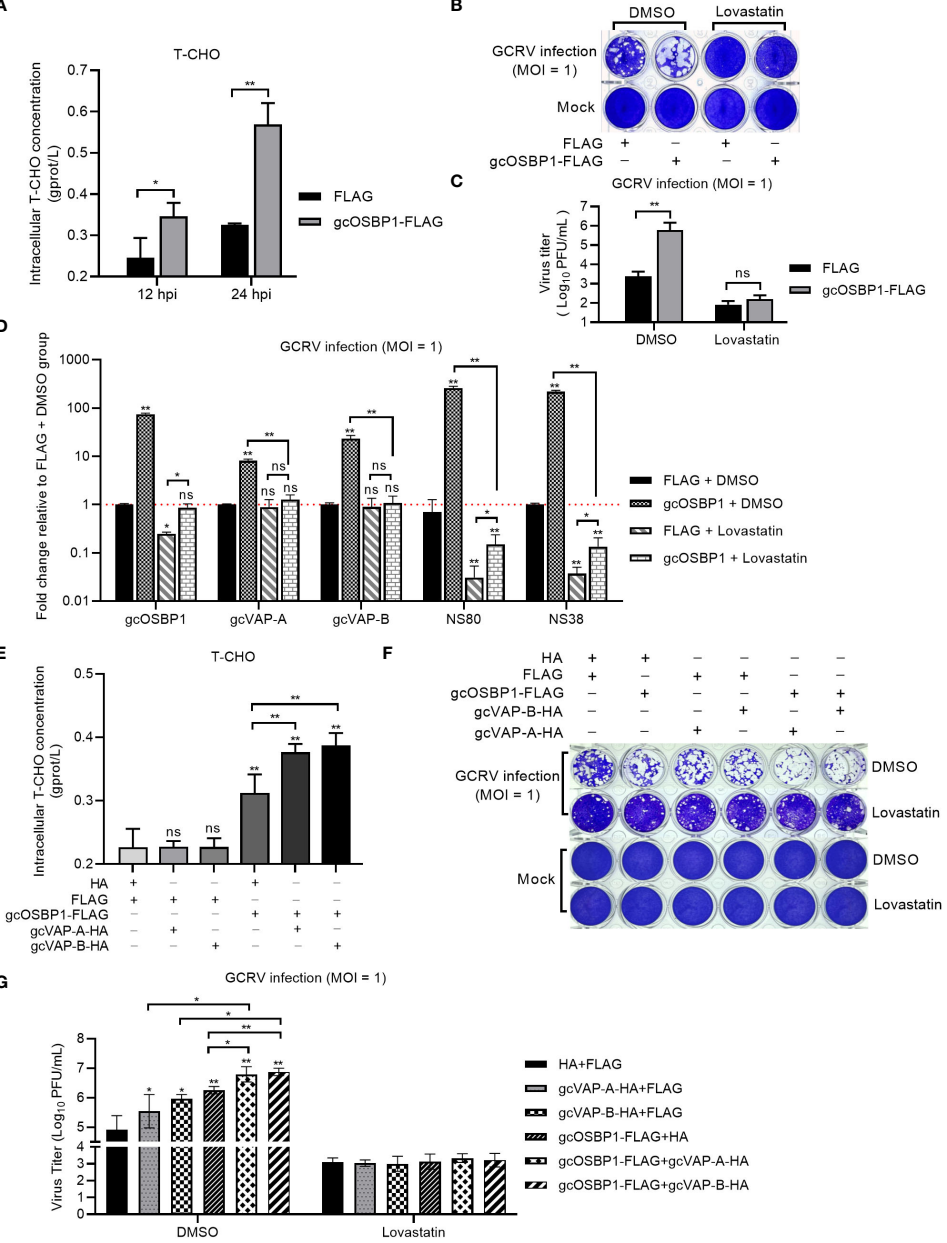
gcOSBP1-mediated cholesterol accumulation is critical for gcOSBP1- and/or gcVAP-A/B-mediated GCRV replication. **(A)** Overexpression of gcOSBP1 increases accumulation of intracellular cholesterol. **(B, C)** Crystal violet staining **(B)** and determination of GCRV titers **(C)** for the effect of inhibiting cholesterol accumulation by lovastatin on the gcOSBP1-mediated GCRV infection. **(D)** The effect of overexpression of gcOSBP1 and/or inhibiting cholesterol accumulation by lovastatin on the expression levels of gcVAP-A, gcVAP-B, NS80 and NS38. **(E)** The effect of overexpression of gcOSBP1 and/or gcVAP-A/B on the accumulation of intracellular cholesterol. **(F, G)** Crystal violet staining **(F)** and determination of GCRV titers **(G)** for the effect of inhibiting cholesterol accumulation by lovastatin on the gcOSBP1- and/or gcVAP-A/B-mediated GCRV replication. For **(D)**, the asterisk above the error bars indicated statistical significance using the group transfected with FLAG plus DMSO as the control group. For **(E, G)**, the asterisk above the error bars indicated statistical significance using the group transfected with FLAG plus HA as the control group. For **(A, C, D, E, G)**, the asterisk above the bracket indicated statistical significance between the two groups connected by the bracket. *p < 0.05; **p < 0.01; ns, not significant.

Our previous study has shown that lowering cholesterol biosynthesis via lovastatin can block the production of VIBs mediated by grass carp vitamin D receptors Vdra/Vdrb (20). Since gcOSBP1 also promoted the numbers of VIBs, we next investigated the effect of cholesterol biosynthesis in the gcOSBP1-mediated promotion of GCRV replication. Cholesterol accumulation was firstly measured in GCRV-infected CIK cells expressing FLAG or gcOSBP1-FLAG. Compared with the control cells expressing FLAG, cholesterol accumulation was observed both at 12 hpi and 24 hpi in the CIK cells expressing gcOSBP1-FLAG (**Figure 6A**). The effect of inhibiting cholesterol accumulation by lovastatin on the gcOSBP1-mediated GCRV infection was investigated. Similar to the results in the untreated cells, overexpression of gcOSBP1 significantly promoted GCRV infection in the cells treated with DMSO. The treatment with lovastatin significantly impaired GCRV infection. Furthermore, overexpression of gcOSBP1 had no impact on GCRV replication in the case of lovastatin treatment (**Figures 6B, C**). Moreover, the results showed that in the cells treated with DMSO, overexpression of gcOSBP1 significantly enhanced the expression of VAP-A, VAP-B, NS80 and NS38 when compared with the FLAG group. The treatment with lovastatin significantly impaired the expression of gcOSBP1, NS80 and NS38, but no effects on the expression of gcVAP-A and gcVAP-B, when compared with the FLAG group treated with DMSO. The treatment with lovastatin also significantly inhibited gcOSBP1-mediated induction of gcVAP-A, gcVAP-B, NS80 and NS38 (**Figure 6D**).

Intracellular cholesterol homeostasis is essential for many viral infections, and mammalian VAP-A and OSBP have been shown to regulate intracellular cholesterol content (24). We also studied the possible effects of gcVAP-A/B and/or gcOSBP1 on the intracellular cholesterol homeostasis. Different from gcOSBP1, overexpression of gcVAP-A or gcVAP-B alone failed to affect intracellular cholesterol content. However, overexpression of gcVAP-A or gcVAP-B significantly strengthened the accumulation of intracellular cholesterol content mediated by gcOSBP1 (**Figure 6E**).

We next tested whether inhibiting cholesterol accumulation by lovastatin affected the synergetic effect of gcOSBP1 and gcVAP-A/B in promoting GCRV infection. Similar to the results in the untreated CIK cells, the cytopathic effects were more obvious in the DMSO-treated CIK cells expressing gcOSBP1 plus gcVAP-A or gcVAP-B compared to CIK cells expressing gcOSBP1, gcVAP-A or gcVAP-B alone (**Figure 6F**). The higher viral titers were also observed in the DMSO-treated CIK cells co-expressing gcOSBP1 plus gcVAP-A or gcVAP-B compared to CIK cells expressing gcOSBP1, gcVAP-A or gcVAP-B alone (**Figure 6G**). However, inhibiting cholesterol accumulation completely abolished the influence of gcOSBP1 and/or gcVAP-A/B in promoting GCRV infection (**Figures 6F, G**).

These results collectively demonstrate that gcOSBP1 but not gcVAP-A or gcVAP-B regulates intracellular cholesterol homeostasis, and that gcOSBP1-mediated cholesterol accumulation is critical for GCRV replication.

### gcOSBP1 synergies with gcVAP-A or gcVAP-B for promoting the generation of VIBs via increasing and binding with more NS38 and NS80 proteins

**Figure 7:**
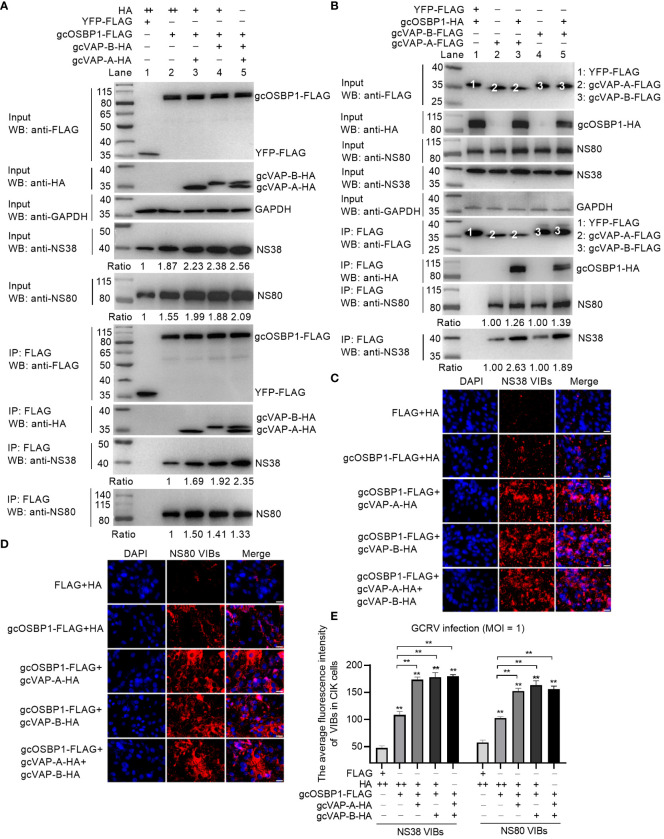
gcOSBP1 synergies with gcVAP-A or gcVAP-B to promote GCRV replication. **(A)** gcVAP-A/B increased the interaction between gcOSBP1 and NS38 or NS80 protein of GCRV. **(B)** gcOSBP1 increased the interaction between gcVAP-A/B and NS38 or NS80 protein of GCRV. **(C)** gcVAP-A/B increased the gcOSBP1-mediated generation of VIBs determined by anti-NS38 antibody. Scale bars, 10 µm. **(D)** gcVAP-A/B increased the gcOSBP1-mediated generation of VIBs determined by anti-NS80 antibody. Scale bars, 10 µm. **(E)** The average fluorescence intensity of gcOSBP1- and/or gcVAP-A/B-mediated generation of VIBs determined by anti-NS38 or anti-NS80 antibody. For **(E)**, the asterisk above the error bars indicated statistical significance using the group transfected with FLAG plus HA as the control group. The asterisk above the bracket indicated statistical significance between the two groups connected by the bracket. **p < 0.01. For **(A, E)**, + indicates that the transfection volume of the plasmid is 0.3 μg, and ++ for 0.6 μg.

Based on the above results (**Figures 4**, **5**) and the finding that gcVAP-A or gcVAP-B cooperates with gcOSBP1 to strengthen the accumulation of intracellular cholesterol content and promote GCRV infection, we further elucidate how gcOSBP1 and gcVAP-A or gcVAP-B coordinate to promote GCRV infection via regulating the generation of VIBs. Since gcOSBP1 is recruited by NS38 and NS80 proteins for promoting the generation of VIBs, we firstly tested whether gcVAP-A or gcVAP-B affected the binding of gcOSBP1 to the NS38 and NS80 proteins of GCRV. As shown in **Figure 7A**, western blotting analysis of input proteins revealed that overexpression of gcOSBP1, gcOSBP1 plus VAP-A, gcOSBP1 plus gcVAP-B, or gcOSBP1 plus gcVAP-A plus gcVAP-B increased the protein levels of NS38 and NS80, when compared to the control group. Results of Co-IP revealed that gcVAP-A/B increased the production of gcOSBP1-NS38 and gcOSBP1-NS80 complexes, especially for gcOSBP1-NS38 complexes (**Figure 7A**). Next, we investigated whether gcVAP-A or gcVAP-B could form complexes with gcOSBP1, NS38 and/or NS80. As shown in **Figure 7B**, Co-IP analysis using FLAG-tagged gcVAP-A or gcVAP-B confirmed that gcOSBP1 was associated with gcVAP-A/B (Lane 3 and 5 using anti-HA antibody for IP product). Similar to gcOSBP1, both gcVAP-A and gcVAP-B interacted with NS38 (Lane 2 and 4 using anti-NS38 antibody for IP product in **Figure 7B**) and NS80 (Lane 2 and 4 using anti-NS80 antibody for IP product in **Figure 7B**). Furthermore, gcOSBP1 increased the production of gcVAP-A/B-NS38 and gcVAP-A/B-NS80 complexes, especially for gcVAP-A/B-NS38 complexes (**Figure 7B**). These results suggest that gcOSBP1, gcVAP-A/B, NS38 and NS80 interact to form complexes.

To explore the effect of gcVAP-A or gcVAP-B in the generation of VIBs exerted by gcOSBP1, the intracellular content of VIBs was examined in immunofluorescence experiments using a specific NS38 or NS80 antibody. Compared with the control cells (FLAG plus HA group), overexpression of gcOSBP1, gcOSBP1 plus gcVAP-A, gcOSBP1 plus gcVAP-B, or gcOSBP1 plus gcVAP-A plus gcVAP-B significantly enhanced the total numbers of VIBs. Compared to the control group (gcOSBP1-FLAG plus HA), gcVAP-A or gcVAP-B further strengthened the numbers of VIBs mediated by gcOSBP1 (**Figures 7C–E**). However, co-overexpression of gcVAP-A and gcVAP-B had no a greater increase in the numbers of VIBs mediated by gcOSBP1 compared with single overexpression of gcVAP-A or gcVAP-B, demonstrating that gcVAP-A and gcVAP-B might play redundant roles in the gcOSBP1-mediated generation of VIBs.

Collectively, these data indicate gcOSBP1 synergies with gcVAP-A or gcVAP-B to increase the expression of NS38 and NS80 proteins of GCRV, strengthen the binding of gcOSBP1 to NS38 and NS80 proteins of GCRV, and facilitate the accumulation of VIBs, thereby resulting in efficient GCRV replication.

## Discussion

We previously have demonstrated that cholesterol and vitamin D receptors Vdra/Vdrb are vital for the generation of VIBs, which are important functional sites for viral replication (20). Grass carp ARF1, a member of the Ras superfamily, is recruited to VIBs, and co-localized with the viral nonstructural protein NS80 and NS38 of GCRV. The activation of grass carp ARF1 promotes the generation of cytoplasmic VIBs and GCRV replication (19). Grass carp TBK1 isoform gcTBK1_tv3 is also recruited by GCRV NS80 and NS38 proteins into VIBs, however impairs the formation of VIBs via the ubiquitination degradations of NS80 and NS38 proteins (16). In this study, we examined the involvement of the gcOSBP1-mediated cholesterol pathway in the replication of GCRV. We found that gcOSBP1 synergized with gcVAP-A or gcVAP-B to modulate the generation of cholesterol and VIBs, thereby promoting GCRV replication.

Mammalian OSBP1 is found in the cytoplasm and ER of wild type H4 human neuroglioma cells, and can be translocated to the Golgi apparatus after treating cells with 25OH (21). Similar to OSBP1 in H4 cells, the translocation of OSBP from a cytoplasmic compartment to the Golgi complex was observed in CHO-K1 cells in the presence of 25OH (25). In Huh7 cells, endogenous OSBP was located in cytoplasmic/vesicular and associated with the Golgi. When 25OH was added, the localization of OSBP was observed mainly in the Golgi (1). These findings suggest that cholesterol regulates the Golgi localization of OSBP. Besides cholesterol, viral infection also regulates the localization of OSBP. Viral replication takes place at the membraneous web (MW), viral replication complexes (VRCs), ROs or VIBs. Under normal conditions, OSBP localized the cytoplasm and Golgi. In cells infected with EMCV, OSBP localization changed and migrated to ER-derived ROs (26). In cells infected with Aichi virus (AiV), OSBP was mainly located at ROs in the perinuclear region and in the vicinity of the ER membrane (27). However the localization of OSBP was not observed in the VRCs of DENV (10). Similar to EMCV and AiV but different from DENV infection, gcOSBP1 localized with virus replication sites VIBs of GCRV via its interaction with the forming proteins of GCRV VIBs including NS80 and NS38, suggesting that gcOSBP1 is directly involved in GCRV replication.

**Figure 8:**
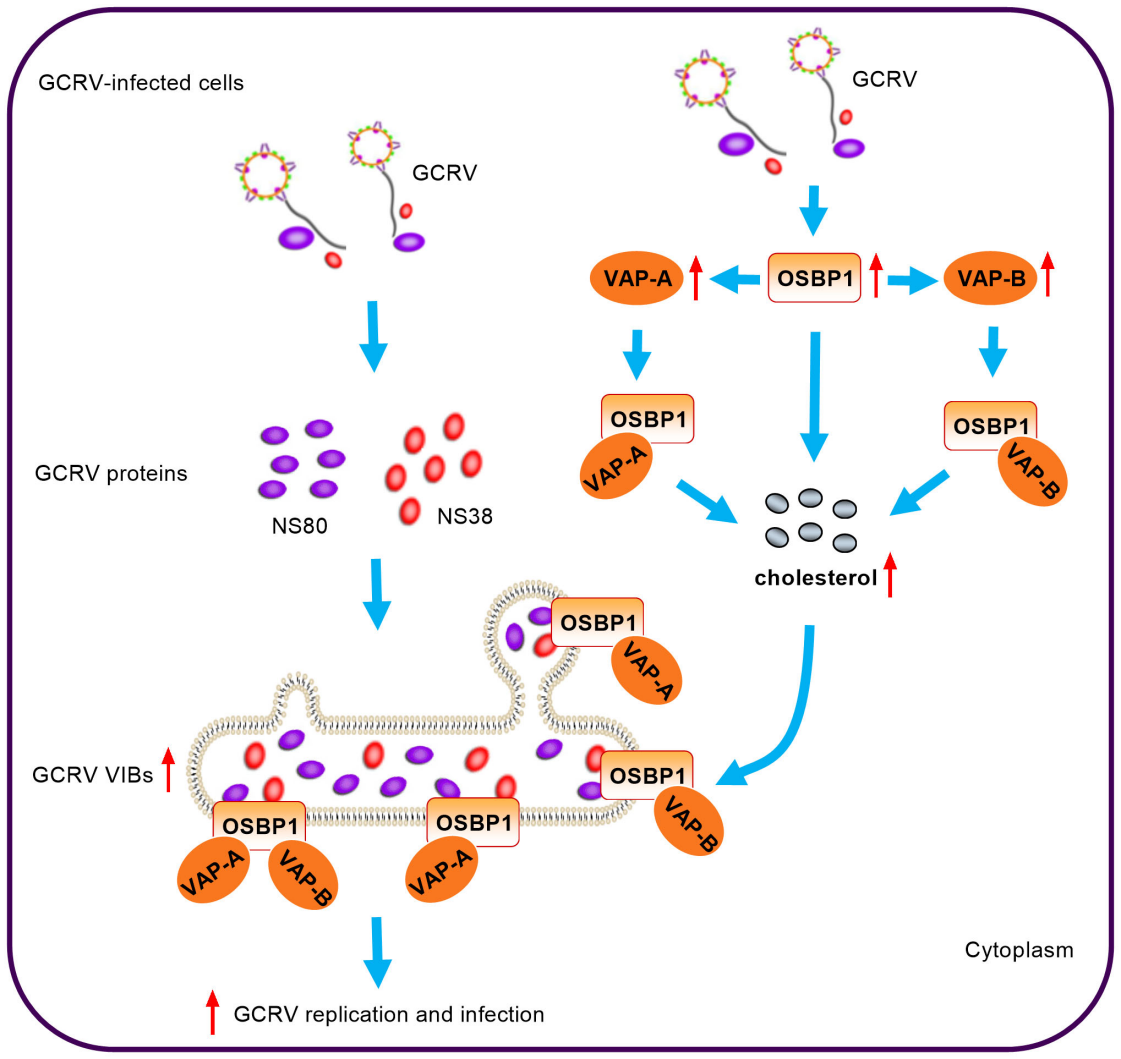
Model for the coordination of gcOSBP1 and gcVAP-A/B to promote grass carp reovirus replication.

VAP-A and its homologue VAP-B have a unique function as specific receptors on the ER, where they anchor proteins to the surface of ER. OSBPs can form protein complexes with VAP-A/B, which facilitate protein, ceramide, cholesterol, or sterol transport at the interface between ER and Golgi apparatus or ROs (28–31). The interaction of VAP-A with OSBP also plays an important role in cholesterol homeostasis and viral infection. Accumulation of cholesterol within late endosomal compartments has been shown to impair the infection of many viruses such as IAV, VSV and DENV that enter the cell through endocytosis. The depletion of VAP-A increased endosomal cholesterol levels, and the disturbance of interaction between VAP-A and OSBP by IFITM3 results in cholesterol accumulation in endosomal compartments, thereby ultimately inhibiting viral entry (24). Another study showed that 25OH and 27-hydroxycholesterol (27OH) disturbed the association of OSBP to VAP-A and induced cholesterol accumulation in the late endosomes, resulting in sequestering viral particles inside the late endosomal compartment thereby preventing cytoplasmic virus replication (32). In the present study, we demonstrated for the first time that gcOSBP1, gcVAP-A and gcVAP-B could promote GCRV replication. Overexpression of gcOSBP1 significantly increased the expression of gcVAP-A/B. Different from these studies mentioned above, we found only gcOSBP1 and gcOSBP1-gcVAP-A/B complexes, but not gcVAP-A/B alone, played an important role in promoting the accumulation of intracellular cholesterol. The gcOSBP1-gcVAP-A/B complexes also contribute to the generation of more VIBs. More importantly, we also reported that cholesterol accumulation was vital for gcOSBP1- and/or gcVAP-A/B-mediated GCRV replication (**Figure 8**). More research is required to understand how gcOSBP1 and gcOSBP1-gcVAP-A/B complexes contribute to cholesterol accumulation and trafficking on VIBs.

PI4P and cholesterol are key lipid components of ROs. In mammals, many viruses including picornaviruses and HCV hijack the OSBP-mediated cholesterol transport pathway in building replication sites, where OSBP is indirectly recruited to the viral RNA replication sites via binding to PI4P (6, 26). In these RNA viruses, including rhinovirus (RV), PV and HCV, the recruitment of OSBP to ROs is dependent on PI4K activity or PI4P (6, 11, 33). For AiV, not only OSBP, but also some components of cholesterol transport pathway such as VAP-A/B and SAC1, are recruited to AiV ROs. However, OSBP is recruited to AiV ROs independently of PI4P (27). RV, PV and AiV belong to picornaviridae. PI4P-independent recruitment of OSBP to AiV ROs and PI4P-dependent recruitment of OSBP to ROs of RV and PV suggest that even among closely related viruses, the processes for hijacking cholesterol transport pathway may be diverse. Besides viruses, the studies have shown that during *Salmonella* Typhimurium infection, OSBP-1 and VAP-A/B are necessary for the stability of *Salmonella*-containing vacuole (SCV), a modified phagosome within which *Salmonella* will survive and replicate (34, 35). Given that both viruses and bacteria require a common host protein OSBP1 to form vacuoles or ROs for pathogen replication, we are interested to know whether gcOSBP1 and its binding partners such as gcVAP-A/B contribute to the generation of GCRV VIBs. Our evidences suggest that GCRV has exploited the common cholesterol transport pathway in assisting virus RNA replication. First, GCRV replication occurs at the VIBs (13). Second, this study showed that gcOSBP1, which mediated cholesterol accumulation and promoted GCRV replication during GCRV infection, could interact with the essential viral proteins NS38 and NS80 that form VIBs. The interaction between gcOSBP1 and NS80 or NS38 of GCRV was suggested to enable the PI4P-independent recruitment of OSBP to viral replication sties. Third, the present study also showed that other components of cholesterol transport pathway such as gcVAP-A/B strengthened the accumulation of intracellular cholesterol content mediated by gcOSBP1 and gcOSBP1-mediated generation of VIBs (**Figure 8**). Together, these observations indicate that although different strategy may be used for recruiting the cholesterol transport system to build replication sites, RNA viruses and even bacteria share critical host components of this pathway.

In conclusion, we identified gcOSBP1, gcVAP-A and gcVAP-B as host factors involved in promoting GCRV replication by targeting the accumulation of intracellular cholesterol content and the generation of VIBs. Though we have focused here on the function of 3 components from cholesterol transport pathway including gcOSBP1 and gcVAP-A/B in GCRV replication, GCRV may hijack other cellular lipid synthesis and trafficking proteins to maintain high cholesterol and PI4P content in the cytoplasm and even in VIBs. More work will be required to investigate the influence of host factors targeting lipid flow on replication membranes and viral replication.

## Data availability statement

The raw data supporting the conclusions of this article will be made available by the authors, without undue reservation.

## Ethics statement

Ethical approval was not required for the studies on animals in accordance with the local legislation and institutional requirements because only commercially available established cell lines were used.

## Author contributions

JL: Conceptualization, Methodology, Writing – original draft, Data curation, Formal analysis, Investigation, Software. JZ: Formal analysis, Methodology, Writing – original draft. YC: Methodology, Writing – original draft. TL: Supervision, Writing – review & editing. MC: Supervision, Writing – review & editing, Conceptualization, Funding acquisition, Methodology, Project administration, Resources, Validation, Writing – original draft.
